# Cannabidiol Effects on Phospholipid Metabolism in Keratinocytes from Patients with Psoriasis Vulgaris

**DOI:** 10.3390/biom10030367

**Published:** 2020-02-28

**Authors:** Iwona Jarocka-Karpowicz, Michał Biernacki, Adam Wroński, Agnieszka Gęgotek, Elżbieta Skrzydlewska

**Affiliations:** 1 Department of Analytical Chemistry, Medical University of Bialystok, Mickiewicza 2d, 15-222 Bialystok, Poland; iwona.jarocka-karpowicz@umb.edu.pl (I.J.-K.); michal.biernacki@umb.edu.pl (M.B.); agnieszka.gegotek@umb.edu.pl (A.G.); 2 Dermatological Specialized Center “DERMAL” NZOZ in Bialystok, 15-453 Bialystok, Poland; adam.wronski@dermal.pl

**Keywords:** psoriasis, cannabidiol, UV, redox balance, phosholipid metabolism

## Abstract

Psoriasis is a chronic inflammatory skin disease characterized by dysregulated keratinocyte differentiation, but oxidative stress also plays an important role in the pathogenesis of this disease. Here, we examined the effect of cannabidiol (CBD), a phytocannabinoid with antioxidant and anti-inflammatory properties, on the redox balance and phospholipid metabolism in UVA/UVB-irradiated keratinocytes isolated from the skin of psoriatic patients or healthy volunteers. CBD accumulates mainly in membrane keratinocytes, especially from patients with psoriasis. This phytocannabinoid reduces the redox imbalance observed in the UV-irradiated keratinocytes of healthy subjects. It does so by decreasing reactive oxygen species (ROS) generation, increasing the Trx-dependent system efficiency, and increasing vitamin A and E levels. Consequently, a reduction in lipid peroxidation products, such as 8-isoprostanes and 4-hydroxynonenal, was also observed. Moreover, CBD modifies redox balance and lipid peroxidation in psoriatic patient keratinocytes following UV-irradiation. Interestingly, these changes are largely in the opposite direction to the case of keratinocytes from healthy subjects. CBD also regulates metabolic changes by modulating the endocannabinoid system that is disturbed by psoriasis development and UV irradiation. We observed a decrease in anandamide level in the UV-irradiated keratinocytes of healthy controls following CBD treatment, while in keratinocytes from patients treated with CBD, anandamide level was increased. However, the level of palmitoylethanolamide (PEA) was decreased in both groups treated with CBD. We further demonstrate that CBD increases CB1 receptor expression, primarily in the keratinocytes of patients, and increases CB2 receptor expression in both the psoriatic and control groups. However, CBD decreases CB2 receptor expression in UV-irradiated keratinocytes taken from patients. The UV- and psoriasis-induced activity of transmembrane transporters (Multidrug-Resistance (MDR) and breast cancer resistance protein (BCRP)) is normalized after CBD treatment. We conclude that CBD partially reduces oxidative stress in the keratinocytes of healthy individuals, while showing a tendency to increase the oxidative and inflammatory state in the keratinocytes of patients with psoriasis, especially following UV-irradiation.

## 1. Introduction

Psoriasis is a chronic inflammatory skin disease. Its pathogenesis involves hyperkeratosis of the epidermis, characterized by inflammatory skin infiltrates, epidermal hypertrophy, and dysregulated keratinocytes proliferation, differentiation, and desquamation [1]. It is believed that the pathogenesis of psoriasis is the result of impaired immunity, possessing a genetic component linked to immune genes and their encoded pathways [2]. In recent years, the dysregulation of these genes, including TNF-α and IL-23, has been shown to participate in the development of psoriasis [3].

The endocannabinoid system—consisting of endocannabinoids, the enzymes responsible for their degradation, and cannabinoid receptors CB1 and CB2—is widely expressed in epidermal cells and plays a vital role in the immune response [4,5,6]. The best-known endocannabinoid is anandamide (AEA), which reduces the levels of various chemokines (including TNF-α and IL-23) in keratinocyte cell lines (HaCaT and NHEK), as well as IL-17 in co-cultures of lymphocytes (Th1 and Th17) and keratinocytes. Importantly, these effects were prevented by a CB1 receptor antagonist [7]. Cannabinoid receptors have been shown to modulate the expression of pro-inflammatory cytokines [8]; the activation of CB1 receptors increases the level of pro-inflammatory cytokines, whereas activation of CB2 receptors promotes their reduction and generation of reactive oxygen species (ROS) [9]. Thus, endocannabinoids can simultaneously modify inflammation and oxidative stress. In addition, oxidative stress resulting from the overproduction of ROS and reduced antioxidant abilities modifies cell signaling pathways, including pro-inflammatory signaling that contributes to the development of psoriasis [10]. However, AEA also reduces keratinocyte differentiation by modifying the activation of the CB1 receptors, which in turn increases the activity of the AEA-degrading enzyme—fatty acid amide hydrolase (FAAH) [11]—and thereby reduces the level of AEA. Another endocannabinoid—palmitoylethanolamide (PEA)—has been demonstrated to be an anti-inflammatory factor that may increase the level of AEA. However, PEA also acts as an activator of transient receptor potential cation channels (TRPVs) and peroxisome proliferator-activated receptors (PPARs) [12,13].

The pharmacological modulation of the endocannabinoid system is a modern approach to regulating inflammation, redox balance, and epidermal functions. It is known that exogenous cannabinoids—through changes in the expression of cannabinoid receptors—could regulate inflammation through various mechanisms, including the reduction in pro-inflammatory cytokines and ROS [14]. Exogenous cannabinoids also influence keratinocyte proliferation and differentiation [14]. It has also been shown that the peroxisome proliferator-activated receptor γ (PPARγ), which is activated by cannabinoids, is expressed in the keratinocytes of psoriasis patients. The topical administration of PPARγ agonists reduces epidermal hyperplasia in experiments in mice and humans [15]. Therefore, the growth and differentiation effect of cannabinoids on human keratinocytes is thought to include mechanisms dependent on cannabinoid receptors, as well as TRPV1 and PPAR, through their ability to reduce inflammation and oxidative stress [16,17].

One of the main phytocannabinoids found in *Cannabis sativa* L. is cannabidiol (CBD), which does not show psychoactivity, but has a wide spectrum of biological activity, including antioxidant, anti-inflammatory, and neuroprotective effects [18]. CBD can regulate cell redox status both directly and indirectly. Direct action leads to a decrease in the oxidative capacity of cells, which is associated with the prevention of ROS generation [19,20]. Additionally, CBD causes an increase in the level of mRNA, as well as the level/activity of antioxidant proteins, including superoxide dismutase (SOD) and glutathione peroxidase (GSH-Px) isoenzymes in various metabolic disorders [21,22]. CBD may also indirectly affect the redox balance by altering cannabinoid receptor activation; CBD can activate/antagonize/inhibit metabotropic receptors in a concentration-dependent manner [23,24]. Consequently, CBD prevents oxidative stress and the oxidative modifications of cellular components (DNA, lipids, and proteins), a feature that can lead to the development of various diseases [18,22].

Despite the evidence that oxidative stress is associated with psoriasis, many of the procedures used to treat psoriasis enhance oxidative stress. For example, in psoralen and UVA treatment, there is mass production of singlet oxygen in the skin [25]. It is known that solar radiation intensifying oxidative stress improves the clinical conditions of patients with psoriasis [26]. It is also believed that UVB phototherapy, often used in psoriasis treatment, causes local exacerbation of oxidative imbalance, but may have a beneficial anti-inflammatory effect on the skin. This may be due to the activation of antiproliferative and proapoptotic pathways in both resident and infiltrative cell populations [27].

Therefore, there is clinical importance for understanding the response of epidermal keratinocytes to CBD, which could represent a novel therapy for psoriasis. This primarily applies to the potential intensification of oxidative stress and, consequently, to metabolic disorders of membrane phospholipids. Such disorders may be accompanied by changes in the activation of membrane receptors that control lipid metabolism, proliferation, differentiation, and the apoptosis of epidermal cells, including keratinocytes. Therefore, the purpose of this study was to assess the effect of CBD on the redox system and phospholipid metabolism induced by UV radiation in cultured keratinocytes isolated from skin of psoriasis patients compared to healthy controls.

## 2. Materials and Methods

Skin tissues were collected from 30 untreated patients with a diagnosis of psoriasis vulgaris and 15 healthy volunteers. The study population included 11 men and 19 women with an age range of 27–54 years (mean age 40) and 6 health men and 9 health women with an age range 28–52 years (mean age 39) forming control group. Eligible patients were diagnosed with plaque psoriasis with at least 10% of their total body surface affected for at least six months. Psoriasis severity was assessed using the Psoriasis Area and Severity Index (PASI) (range 10–25; median 17). None of the patients or healthy subjects received topical, oral, or injectable medications for 4 weeks prior to the study. Patients with a history indicating the presence of other disorders were excluded from the study. None of the study participants were smokers and or excessive consumers of alcohol. The study was conducted in accordance with the Declaration of Helsinki, and the protocol was approved by the Local Bioethics Committee of the Medical University of Bialystok (Poland), No. R-I-002/289/2017. Written informed consent was obtained from all participants.

Skin fragments immediately after biopsy were sent for histopathological examination (hematoxylin-eosin staining). The rest of the sample was washed in PBS with 50 U/mL penicillin and 50 μg/mL streptomycin and was incubated overnight in dispase (1 mg/mL) at 4 ^°^C to separate the epidermis from the dermis. Following incubation, the epidermis was digested for 20 min at 37 °C using trypsin/EDTA. Separated keratinocytes were washed and resuspended in cell culture medium.

### 2.1. Cell Culture and Treatment

Obtained keratinocytes were cultured in a Keratinocyte Growth Kit (Gibco, Grand Island, NY, USA) containing Keratinocyte Serum-Free Medium suplemented with 10% fetal bovine serum, 5 µg/L epidermal growth factor EGF 1–53, 50 U/mL penicillin, and 50 μg/mL streptomycin. The cells were cultured under standard conditions (humidified atmosphere of 5% CO_2_ at 37 °C) until the 4^th^ passage. When keratinocytes reached 80% confluence, they were washed with PBS (37 °C) and exposed to UV radiation in PBS (4 °C) to avoid heat stress and oxidation of the medium components. The 70% cell viability measured by the MTT assay [28] was an indicator of the doses selection. The cells were irradiated on ice at a distance of 15 cm from the 6 lamps (Bio-Link Crosslinker BLX 365/312; Vilber Lourmat, Germany) assembly at 6 W each, corresponding to 4.2 mW/cm^2^ and 4.08 mW/cm^2^, for UVA (365 nm) and UVB (312 nm), respectively. The total radiation doses were as follows: UVA-30 J/cm^2^ and UVB-60 mJ/cm^2^.

The effect of CBD on keratinocytes was examined as follows: all cell groups (control cells, keratinocytes following UVA irradiation, and cells following UVB irradiation) were cultured for 24 h in a medium suplemented with 4 µM CBD (dissolved in ethanol with a final concentration of 0.1%). This concentration of CBD did not alter the morphology or proliferation of keratinocytes [29,30], nor did it affect the cells’ viability measured by an MTT assay [28] (data not shown). The control cells were cultured in parallel without treatment. After incubation, all cells were washed with PBS, collected by scraping into cold PBS, and centrifuged. The cells were then resuspended in PBS and sonificated. A Bradford assay was used for total protein content measuring [31].

### 2.2. Methods

#### 2.2.1. Analysis of Oxidation

NADPH oxidase (NOX-EC 1.6.3.1) activity was measured by the luminescent method with lucigenin as luminophore. The enzyme amount required to release 1 nmol of O^2-^ per minute was described as one unit of NOX activity, and is presented in relative luminescence units (RLU) per milligram protein [32].

Xanthine oxidase (XO-EC1.17.3.2) activity was measured according to the method published by Prajda and Weber [33]. The changes in absorbance at 290 nm was measured for uric acid formation from the xanthine substrate detection. One unit of XO was defined as the amount of the enzyme, which is required to release 1 μM of uric acid per minute. The enzyme-specific activity is described in microunits per milligram of protein.

The generation of reactive oxygen species (ROS) was detected using the selective interaction of the spin probes, CMH (1-hydroxy-3-methoxy-carbonyl-2,2,5,5-tetrame-thylpyrrolidine, 200 µM), with ROS. The resulting complexes were measured by an electron spin resonance (ESR) spectrometer e-scan (Noxygen GmbH/Bruker Biospin GmbH, Germany) [34]. The ROS generation is expressed in micromoles per minute per milligram of protein.

#### 2.2.2. Analysis of Antioxidants

Glutathione peroxidase (GSH-Px-EC.1.11.1.6) activity was measured spectrophotometrically using the method described by Paglia and Valentine [35]. The oxidation of 1 µmol NADPH min^−1^ at 25 °C and pH 7.4 was defined as one GSH-Px activity unit and normalized per protein concentration in each sample. Glutathione (GSH) was quantified using capillary electrophoresis (CE) with UV detection at 200 [36]. The GSH concentration was determined from the calibration curve over a range: 1–120 nmol/L (r^2^ = 0.9985) and the level of GSH is expressed as the nanomoles per milligram of protein.

Thioredoxin reductase (TrxR-EC.1.8.1.9) activity was assessed basing on the NADPH-mediated reduction of 5,5′-dithiobis(2-nitrobenzoic) acid (DTNB) to yellow 5-thio-2-nitrobenzoic acid (TNB) [37]. Measurements were made using reagents from the commercially available kit (Sigma-Aldrich, St. Louis, MO, USA; CN CS0170). One unit of TrxR was defined as the amount of the enzyme, which caused an increase in A_412_ of 1.0 per min per mL at 25 °C and pH 7.0. The enzyme-specific activity is expressed in units normalized per milligram of protein.

The thioredoxin (Trx) level was quantified using the ELISA method [38]. Standards and cell lysates were loaded into ELISA plate wells (Nunc Immuno Maxisorp, Thermo Scientific, Waltham, MA, USA) and incubated overnight at 4 °C with an anti-thioredoxin primary antibody (Abcam, Cambridge, MA, USA). After washing, the plates were incubated at room temperature (RT) for 30 min with a peroxidase blocking solution (3% H_2_O_2_, 3% fat-free dry milk in PBS). Then, as a secondery antibody, goat anti-rabbit secondary antibody (Dako, Carpinteria, CA, USA) was added for 1 h at RT. Next, for 40 min, chromogen substrate solution (0.1 mg ml^−1^ TMB, 0.012 % H_2_O_2_) was added to each well. The reaction was stopped by sulfuric acid. Absorption was read at 450 nm with the reference filter set to 620 nm. The level of thioredoxin is expressed in micrograms per milligram of protein.

Catalase (CAT-EC.1.11.1.9) activity was assessed spectrophotometrically (at 240 nm) by measuring the rate of hydrogen peroxide decomposition, using a previously published method [39]. The amount of the enzyme catalyzing the decomposition of 1 µmol of hydrogen peroxide to water and oxygen within 1 min was described as one unit of CAT activity. The enzyme-specific activity is expressed in units per milligram of protein.

Vitamin A and E levels were detected using high-performance liquid chromatography (HPLC) [40]. The vitamins were extracted from cell lysates using hexane, which was dried and diluted in ethanol. A total of 50 µL of the mixture was injected into the RP-18 column. The flow rate was 1 mL/min of methanol and water (95:5). UV detection at 294 nm was applied. The vitamin’s concentration was determined using a calibration curve range: 0.125–1 mg/L for vitamin A (r^2^ = 0.9998) and 5–25 mg/L for vitamin E (r^2^ = 0.9999). The levels of vitamins are expressed as micrograms per milligram of protein.

#### 2.2.3. Analysis of Lipid Peroxidation Products

Lipid peroxidation was estimated by measuring the level of 8-isoprostanes and 4-hydroxynonenal (4-HNE). The total 8-isoprostanes (8-isoPGF_2α_) level was determined, after solid phase extraction, based on the method from Coolen et al. [41], using ultra-performing liquid chromatography tandem mass spectrometry (LCMS 8060, Shimadzu, Kioto, Japan). A Zorbax Eclipse Plus C18 column (2.1 mm × 100 mm, 1.8-micron) was employed. Gradient separation was performed, starting with a solvent that consisted of 60% H_2_O (pH 3.85) and 40% acetonitrile (ACN) running for 4 min. In 1 min, a gradient was run to 100% ACN, which ran until 8 min. From 8 to 9 min, a gradient was used to return to the original eluent composition and the system was equilibrated until 15 min. Due to the presence of a carboxylic group, electrospray ionization (ESI) operating in negative ion mode with multiple-reaction monitoring (MRM) mode provided the best sensitivity. For MRM analysis, the mass transitions m/z 353.2 → 193.1 for 8-isoPGF_2α_ and 357.2 → 197.1 for 8-isoPGF_2α_-d_4_ were selected. The level of 8-isoPGF_2α_ is expressed in the picograms per milligram of protein.

4-HNE levels were measured as O-PFB-oxime-TMS derivatives using minor modifications of the method from Tsikas et al. [42]. Gas chromatography mass spectrometry (GCMS) was used in the selected ion monitoring (SIM) mode. To each sample, 50 pmoL of 4-HNE-d_3_ as an internal standard was added. Aldehyde was derivatized by the addition of O-(2,3,4,5,6-pentafluorobenzyl) hydroxylamine hydrochloride (PFBHA·HCl) (5 mg/mL in PIPES buffer) and N,O-bis(trimethylsilyl)trifluoroacetamide in 1% trimethylchlorosilane (BSTFA:TMCS; 99:1) to form TMS ethers. Derivatized aldehyde was analyzed using a 7890A GC-7000 quadrupole MS/MS (Agilent Technologies, Palo Alto, CA, USA) equipped with a HP-5ms capillary column (0.25 mm id., 30 m length). The column temperature, initially set at 50 °C for 1 min, was raised as follows: 10 °C/min until 200 °C, 3 °C/min until 220 °C, 20 °C/min until 310 °C, and maintained at 310 °C for 5 min. The injector temperature was maintained at 250 °C, the transfer line was kept at 280 °C, and the source temperature was set to 230 °C. Derivatized aldehydes were detected using the single ion monitoring (SIM) mode. The ion used for 4-HNE identification was as follows: m/z 242 for 4-HNE-PFB-TMS, and m/z 245,0 for IS derivative (4-HNE-d_3_-PFB-TMS). The level of 4-HNE is expressed as nanomoles per milligram of protein.

#### 2.2.4. Measurement of 4-HNE-protein Adduct Levels

The level of 4-HNE-protein adducts was measured using the ELISA method described above (thioredoxin level measurement), using an anti-4-HNE-His antibody (genuine anti-4-HNE-His murine monoclonal antibody, clone 4-HNE 1g4) and goat anti-mouse antibody (Dako, Carpinteria, CA, USA) [43]. The results are presented as a percentage of the expression determined in control cells.

#### 2.2.5. Analysis of Cannabinoid Levels

The level of the endocannabinoids: arachidonoylethanolamine (anandamide, AEA), 2-arachidonoyloglycerol (2-AG), and palmitoylethanolamide (PEA) was measured using ultra-performing liquid chromatography tandem mass spectrometry (LCMS 8060, Shimadzu, Kioto, Japan) [44]. A Poroshell 120 EC-C18 column (3.0 mm × 150 mm, 2.7-micron) was employed. The initial chromatographic conditions were 70% of ACN in water containing 0.1% (v/v) of formic acid as an ionizing agent. After isocratic development for 1 min, a gradient was applied up to 80% ACN from min 1–5, followed by a second gradient up to 88% ACN from min 5–15; then, 100% ACN was reached after 0.5 min. These conditions were kept constant until the end of the chromatographic step that finishes at min 25. Endocannabinoids were extracted using solid phase extraction (SPE) and were analyzed in the positive-ion mode (MRM). AEA-d_8_, 2-AG-d_8_, and OEA-d_4_ were used as internal standards for quantification. The precursor to the product ion transition was as follows: m/z 348.3 → 62.15 for AEA, m/z 379.3 → 287.25 for 2-AG, and 300.3 → 62.00 for PEA. Endocannabinoid levels are expressed in fmoles normalized per milligram of protein.

The level of CBD in the cytosol and cell membrane was determined using ultra-performing LCMS (LCMS 8060, Shimadzu, Kioto, Japan) [44]. Cell lysates obtained by sonification were centrifuged (15,000 × *g* 10 min, 4 °C) and the supernatant containing the cytosolic fraction was separated from the membranes fraction. CBD from samples was extracted using SPE, separeted on Poroshell 120 EC-C18 column (3.0 mm × 150 mm, 2.7-micron) as decribed above, and analyzed in the positive-ion mode (MRM). CBD-d_9_ was used as an internal standard for quantification. The precursor to the product ion transition was as follows: 315.1 → 193.00 for CBD. The level of CBD was expressed in micrograms per milligram of protein.

#### 2.2.6. Measuring the Activity of Endocannabinoid-Degrading Enzymes

The activity of enzymes involved in phospholipid metabolism—fatty acid amide hydrolase (FAAH-EC-3.5.1.99) and monoacylglycerol lipase (MAGL-EC 3.1.1.23)—was examined as follows: FAAH activity was assessed by a spectrophotometric measurement of the level of m-nitroaniline (m-NA) released from m-nitroaniline decanol (at 410 nm) [45], while MAGL by spectrophotometric measurement (at 412 nm) of 5’-thio-2-nitrobenzoic acid formation during a 1 min reaction [46]. Enzymatic activity is expressed as nanomoles of the m-NA per minute per milligram of protein.

#### 2.2.7. Measuring Protein Expression

A Western Blot analysis of cellular protein was performed according to Eissa and Seada [47]. Whole-cell lysates or membrane fractions (from 6 randomly selected from healthy and psoriatic subjects) were mixed with a Laemmle buffer containing 5% 2-mercaptoethanol, heated at 95 °C for 10 min, and separated by 10% Tris-Glycine SDS-PAGE. The separated proteins were transferred from the gels onto a nitrocellulose membrane, which was blocked for 1 h with 5% skim milk. Primary monoclonal antibodies (host: rabbit) raised against CB1/2, TRPV1 (Santa Cruse Biotechnology, Santa Cruz, CA, USA), as well as alcaline phophatase labeled secondary anti-rabbit antibodies (Sigma-Aldrich, St. Louis, MO, USA) were used at a concentration of 1:1000. Protein bands were visualized using a commercially available BCIP/NBT Liquid substrate system (Sigma-Aldrich, St. Louis, MO, USA) and were quantitated using the Versa Doc System and Quantity One software (Bio-Rad Laboratories Inc., CA, USA). The level of proteins is expressed as the percentage of the value determined from the control cells.

#### 2.2.8. Membrane Transporter Activity

ABC transporters’ activity was determined using a Multidrug-Resistance (MDR) assay according to the manufacturer’s protocols (eFluxx-ID Multidrug resistance assay kits, Enzo LifeSciences, UK) [48]. All samples were mixed with specyfic for transporter inhibitors (MDR1 inhibitor—Verapamil, MRP inhibitor—MK-571, BCRP inhibitor—Novobiocin in DMSO), and without inhibitors as a control (containing PBS and DMSO). The samples were then incubated for 5 min at 37 °C in dark in 96 well plates. Next, an EFLUXX-ID^®^ green detection reagent was added to the wells. Luminescence was measured at excitation λ = 485 nm and emission λ = 535 nm in En Spire 2300 Multilable Reader (Perkin Elmer). The activity of the enzymes is expressed as a percentage of the value determined from the control cells.

### 2.3. Statistical Analysis

The results obtained in the current study were expressed as the mean ± standard deviation (SD) for n = 30 (psoriatic patients) and n = 15 (healthy volunteers). The data were analyzed using standard statistical analyses, including one-way analysis of variance (ANOVA) with Tukey’s test for multiple comparisons to determine significant differences between different groups. *p*-values less than 0.05 were considered significant.

## 3. Results

### 3.1. Effect of CBD on Redox Imbalance in UV-Irradiated Keratinocytes of Psoriatic Patients

**Figure 1:**
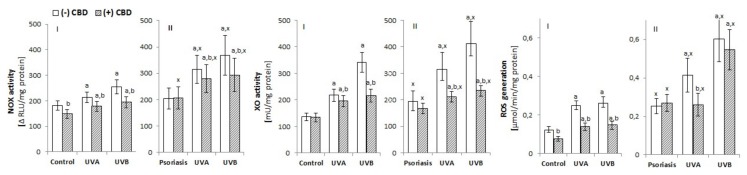
Effect of cannabidiol (CBD) (4 µM) on NADPH oxidase and xanthine oxidase (XO) activity as well as reactive oxygen species (ROS) generation in keratinocytes exposed to UVA (30 J/cm^2^) and UVB (60 mJ/cm^2^) radiation. The keratinocytes were obtained from healthy subjects (**I**) (n = 15) and patients with psoriasis vulgaris (**II**) (n = 30). The mean values ± SD are presented with statistically significant differences: ^a^-vs. control/psoriasis group; ^b^-vs. group without CBD; ^x^-psoriasis vs. control groups; *p* < 0.05.

To assess the effect of psoriasis on the redox balance of the epidermis, ROS generation and the activity/level of enzymatic and non-enzymatic antioxidants were determined in keratinocytes from patients with psoriasis vulgaris. Keratinocytes from patients with psoriasis were characterized by a higher activity of enzymes responsible for the cellular generation of superoxide anion (NOX, XO). Consequently, these keratinocytes also showed increased ROS generation compared with cells from healthy controls (Figure 1). Additionally, exposure of keratinocytes to UVA/UVB radiation increased NOX and XO activity and ROS levels in both groups, especially in cells of patients with psoriasis, where the levels were further increased. CBD treatment was demonstrated to partially prevent the disturbances in keratinocyte redox balance observed in psoriasis patients as well as following UV radiation (Figure 1). However, in the case of keratinocytes from patients with psoriasis, CBD only prevented the increase of ROS levels under the influence of UV radiation. It is noteworthy that CBD clearly reduced ROS level in keratinocytes from healthy individuals, both non-irradiated and irradiated.

**Figure 2:**
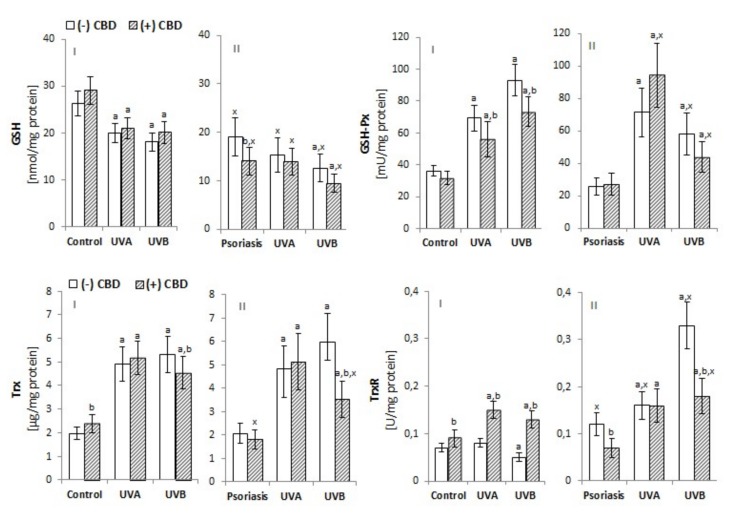
Effect of CBD (4 µM) on reduced glutathione (GSH) level (A-I and A-II) and glutathione peroxidase (GSH-Px) activity as well as thioredoxin (Trx) level and thioredoxin reductase (Trx-R) activity in keratinocytes exposed to UVA (30 J/cm^2^) and UVB (60 mJ/cm^2^) radiation. Keratinocytes were obtained from healthy subjects (**I**) (n=15) and patients with psoriasis vulgaris (**II**) (n = 30). The mean values ± SD are presented with statistically significant differences: ^a^-vs. control/psoriasis group; ^b^-vs. group without CBD; ^x^-psoriasis vs. control groups; *p* < 0.05.

**Figure 3:**
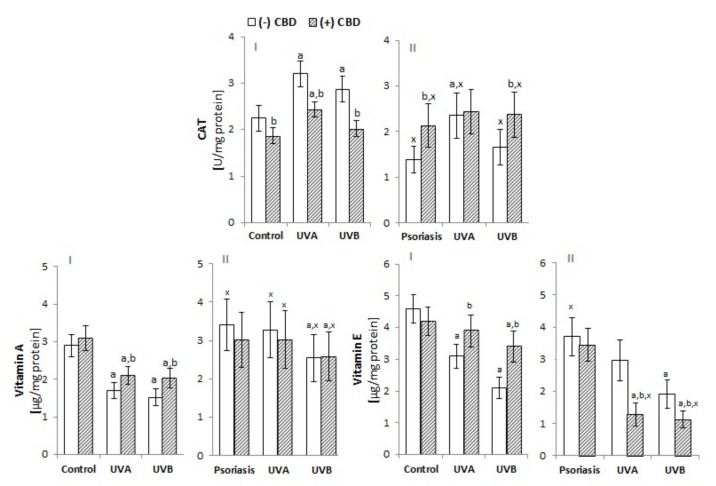
Effect of CBD (4 µM) on catalase activity as well as vitamin A and vitamin E levels in keratinocytes exposed to UVA (30 J/cm^2^) and UVB (60 mJ/cm^2^) radiation. The keratinocytes were obtained from healthy subjects (**I**) (n = 15) and patients with psoriasis vulgaris (**II**) (n = 30). The mean values ± SD are presented with statistically significant differences: ^a^-vs. control/psoriasis group; ^b^-vs. group without CBD; ^x^- psoriasis vs. control groups; *p* < 0.05.

The development of psoriasis and exposure to UV radiation are both factors that modify the level/activity of antioxidants and their receptors in keratinocytes. The level of GSH and the activity of GSH-Px in the keratinocytes of psoriasis patients were both significantly reduced, and UV irradiation intensified these changes (Figure 2). However, the level of Trx was unchanged between patients and controls, and Trx-R activity was higher in keratinocytes from psoriasis patients. UV irradiation increased Trx levels in keratinocytes from healthy and patient cells, whereas TrxR activity was only increased in keratinocytes from patients. The activity of catalase, another enzyme responsible for phospholipid protection, was enhanced in keratinocytes from patients compared with controls (Figure 3). However, the direction of the changes in catalase activity after UV irradiation was similar to GSH-Px activity, while changes in the levels of lipophilic antioxidants such as vitamin E and A followed a similar trend to the main hydrophilic antioxidant—GSH (Figure 2). CBD treatment tended to increase GSH and Trx levels in keratinocytes from healthy and UV-irradiated individuals, whereas it decreased the levels of these compounds in patient keratinocytes. In the case of GSH-Px, in the keratinocytes of healthy individuals, CBD tended to decrease activity, whereas in keratinocytes from patients and UVA-irradiated samples, CBD increased activity of this enzyme. However, the activity of TrxR was increased in the keratinocytes of healthy individuals after CBD treatment but was decreased in the keratinocytes of patients and UVB-irradiated samples. The activity of CAT showed changes in direction similar to GSH-Px (Figure 3). CBD partially prevented the reduction of both vitamins in keratinocytes from healthy subjects exposed to UV, while it tended to reduce the level of both vitamins in keratinocytes from psoriasis patients. CBD reduced the redox imbalance observed in UV-irradiated keratinocytes of healthy individuals. It did so by reducing ROS generation, increasing the Trx-dependent system’s capabilities, and increasing the levels of vitamins A and E. The obtained results indicate that CBD reduces the redox imbalance observed in the UV-irradiated keratinocytes of healthy individuals.

### 3.2. Effect of CBD Phospholipid Metabolism in the UV-Irradiated Keratinocytes of Psoriatic Patients

#### 3.2.1. Lipid Peroxidation

**Figure 4:**
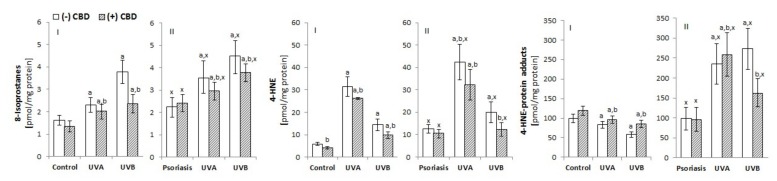
The effect of CBD (4 µM) on lipid peroxidation products, such as 8-isoprostanes and 4-HNE, as well as 4-HNE-protein adduct levels in keratinocytes exposed to UVA (30 J/cm^2^) and UVB (60 mJ/cm^2^) radiation. The keratinocytes were obtained from healthy subjects (**I**) (n = 15) and patients with psoriasis vulgaris (**II**) (n = 30). The mean values ± SD are presented with statistically significant differences: ^a^-vs. control/psoriasis group; ^b^-vs. group without CBD; ^x^-psoriasis vs. control group; *p* < 0.05.

The shift in the redox balance towards oxidative conditions, as a consequence of psoriasis or UV irradiation, altered ROS-dependent and enzyme-dependent phospholipid metabolism. The keratinocytes of patients with psoriasis were characterized by a significantly higher level of ROS-dependent lipid peroxidation products, including, approximately, a two-fold increase in 4-HNE (an aldehyde product of phospholipid fragmentation) and a 30% increase in 8-isoprostanes (the product of phospholipid cyclization) (Figure 4). Consequently, an increased level of 4-HNE-protein adducts was observed. UVA irradiation increased the level of the 4-HNE and its protein adducts to a greater degree in the keratinocytes of healthy people (5-fold) compared to psoriatic patients (3.5-fold); UVB irradiation increased the level of 4-HNE by approximately 2.5-fold in healthy keratinocytes and 1.5-fold in psoriatic patients. CBD treatment reduced the level of 8-isoprostanes as well as 4-HNE and 4-HNE-protein adducts in UV-irradiated keratinocytes and had a stronger effect on the cells from patients compared to the cells of healthy people.

#### 3.2.2. Enzymatic Phospholipid Metabolism

**Figure 5:**
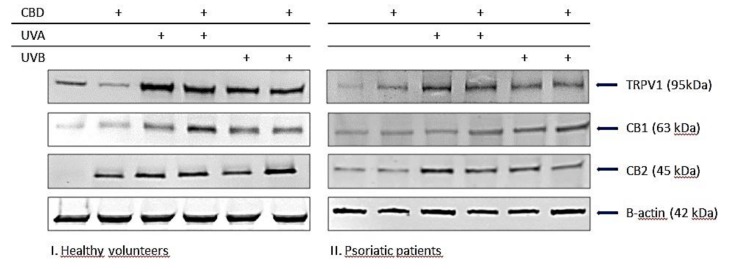
Effect of CBD (4µM) on endocannabinoid system components: endocannabinoids (anandamide (AEA), 2AG, and palmitoylethanolamide (PEA)), enzymes degrading endocannabinoids (fatty acid amide hydrolase (FAAH) and monoacylglycerol lipase (MAGL)), and receptors activated by lipid mediators (CB1, CB2, TRPV1) in keratinocytes exposed to UVA (30 J/cm^2^) and UVB (60 mJ/cm^2^) radiation. The keratinocytes were obtained from healthy subjects (n = 15 for endocannabinoids and degrading enzymes; n = 6 for CB1/2 and TRPV1) (**I**) and patients with psoriasis vulgaris (n = 30 for endocannabinoids and degrading enzymes; n = 6 for CB1/2 and TRPV1) (**II**) The mean values ± SD are presented with statistically significant differences: ^a^-vs. control/psoriasis group; ^b^-vs. group without CBD; ^x^-psoriasis vs. control group; *p* < 0.05.

The consequence of oxidative stress, which is observed both in the manifestation of psoriasis as well as after UV irradiation, is an intensified enzyme-dependent phospholipid metabolism. Some of the products of this modified metabolism are endocannabinoids. The levels of two basic endocannabinoids—AEA and 2-AG—were reduced in keratinocytes from psoriasis patients, whereas the level of the atypical endocannabinoid PEA was increased (Figure 5). UV radiation, especially UVB, also decreased AEA and 2-AG, and significantly increased PEA levels; these changes were significantly greater in keratinocytes from psoriatic patients compared to healthy individuals. CBD counteracted both the reduction of AEA in keratinocytes from healthy individuals, as well as the increase in the level of PEA in psoriatic keratinocytes (both with and without UV irradiation).

Changes in endocannabinoid levels were accompanied by changes in both the activity of the enzymes responsible for their degradation and the expression of membrane receptors, which are ligands. An increase in FAAH and MAGL activity was observed in patient keratinocytes, while the UV exposure of cells increased the activity of these enzymes, particularly in patient keratinocytes. In contrast, CBD treatment mainly decreased MAGL activity, especially in irradiated keratinocytes from patients with psoriasis. The presence of psoriasis, as well as the irradiation of keratinocytes with UVA/B radiation, was accompanied by an increased expression of the receptors tested (CB1/2 and TRPV1). CBD treatment increased CB1 receptor expression in keratinocytes from patients, as well as in UV-irradiated keratinocytes from both groups (healthy and patient cells). CBD increased the expression of CB2 receptors in healthy keratinocytes and patients, while significantly reduced CB2 expression in UV-irradiated keratinocytes from patients. CBD also increased TRPV1 receptor expression, but only in the healthy group and patients with psoriasis without UV exposure. The results of the endocannabinoid system highlight the antioxidant effect of CBD on keratinocytes from healthy individuals.

#### 3.2.3. The Activity of Membrane Transporters in Keratinocytes

**Figure 6:**
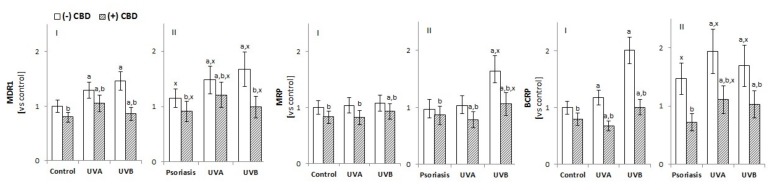
Effect of CBD (4 µM) on the multidrug resistance protein 1 (MDR1), multidrug resistance protein (MRP), and breast cancer resistance protein (BCRP) transporters in keratinocytes exposed to UVA (30 J/cm^2^) and UVB (60 mJ/cm^2^) radiation. The keratinocytes were obtained from healthy subject (n = 15) (**I**) and patients with psoriasis vulgaris (n = 30) (**II**) The mean values ± SD are presented with statistically significant differences: ^a^-vs. control/psoriasis group; ^b^-vs. group without CBD; ^x^-psoriasis vs. control group; *p* < 0.05.

The transfer of exogenous and endogenous substances across cell membranes depends largely on the activity of ABC membrane transporters, such as multidrug resistance protein 1 (MDR1/ABCB1), multidrug resistance protein (MRP/ABCC), and breast cancer resistance protein (BCRP/ABCG2). An increase in MDR1 and BCRP activity was observed in keratinocytes of psoriatic patients and cells of both groups after UVA/B irradiation (Figure 6). In contrast, the treatment of cells with CBD inhibited the activity of all assessed transporters, with BCRP activity in the keratinocytes of patients with psoriasis and irradiated with UVA/B exhibiting the most robust response.

#### 3.2.4. The Penetration of Keratinocytes by CBD

**Figure 7:**
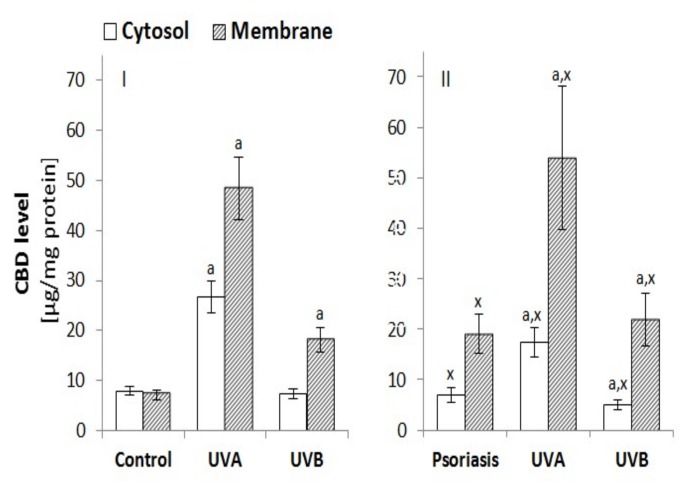
Effect of CBD (4 µM) on this phytocannabinoid level in cytosol and the membranes of keratinocytes exposed to UVA (30 J/cm^2^) and UVB (60 mJ/cm^2^) radiation. The keratinocytes were obtained from healthy subject (n = 15) (**I**) and patients with psoriasis vulgaris (n = 30) (**II**) The mean values ± SD are presented with statistically significant differences: ^a^-vs. control/psoriasis group; ^x^-psoriasis vs. control group; *p* < 0.05.

Changes in the metabolism of membrane phospholipids and in the activity of membrane transporters caused by the effects of psoriasis and UV radiation are accompanied by changes in the way CBD penetrates the keratinocytes (Figure 7). The treatment of keratinocytes from healthy individuals with CBD resulted in its absorption into the membrane and cytosol at similar levels. However, in the case of keratinocytes from patients with psoriasis, cell membranes accumulated as much as 3 times more CBD than the cytosol. UV radiation also changed the level of CBD in the cytosol and membrane. In the case of keratinocytes from healthy individuals, UVA radiation increased CBD levels in the cytosol almost 3-fold, and by approximately 5-fold in membranes, whereas UVB radiation increased CBD approximately only 2-fold in keratinocyte membranes. However, in the case of keratinocytes from patients with psoriasis, cell membranes accumulated more than 2 times more CBD than the cytosol. In contrast, UVA radiation increased the absorption of CBD to the cytosol by about 2-fold and to membranes by about 2.5-fold. UVB radiation tended to decrease the cytosol CBD level and produce about a 3-fold increase in membrane CBD level. As a consequence, both psoriasis and UV radiation increased CBD accumulation in the keratinocyte membrane.

## 4. Discussion

### 4.1. Redox Conditions in Psoriatic Patient Keratinocytes Treated with CBD

Skin cells are often exposed to reactive oxygen species (ROS), which arise as a result of environmental factors, but also in response to changes in cellular metabolism resulting from the development of skin diseases, including psoriasis [10]. Although endogenous antioxidants counteract the effects of ROS overproduction, the excessively high and/or long-lasting presence of ROS in cells, such as in disease conditions, leads to oxidative stress. Recently, we have shown a shift in redox balance towards oxidative conditions with the formation of oxidative stress in plasma, lymphocytes, and granulocytes of patients with psoriasis, leading to the intensification of oxidative modifications of lipids, proteins, and other endogenous compounds [49,50,51]. Different populations of activated leukocytes infiltrating skin lesions also lead to an increase in the level of local oxidants and cause a pro-inflammatory response, as well as oxidative modifications of proteins and lipids in skin cells, especially in keratinocytes [52,53]. Despite the evidence that oxidative stress occurs systemically and locally in psoriasis, many current treatments, to accelerate the removal of damaged epidermal cells, further increase oxidative stress, leading to their death [54]. This is particularly important for skin exposed to UV radiation, which leads to the mass production of singlet oxygen [25].

Ongoing research has confirmed the existence of a redox imbalance in keratinocytes of patients with psoriasis, resulting from both increased ROS production and the dysregulation of GSH- and Trx-dependent antioxidant systems. Additionally, a stronger pro-oxidative response to UV radiation has been observed in keratinocytes from patients with psoriasis compared to healthy controls. Both GSH and Trx can exist in reduced or oxidized forms, thus participating in maintaining the proper redox state of cells by reducing the disulfide bridges of peptides and proteins [55]. However, the results obtained here indicate that the GSH-dependent system in psoriatic keratinocytes is not fully effective, as GSH level is reduced as a result of disease development and is further decreased after UV irradiation. This may result from an increased level of membrane transporters, especially MRP1 [56,57], but could also be a consequence of the formation of GSH conjugates with 4-HNE [58], the level of which is increased in the keratinocytes psoriasis patients. GSH—with glutathione peroxidase—participates in the protection of membrane phospholipids. Therefore, the reduced level of this tripeptide as a peroxidase co-substrate prevents the reduction in lipid peroxides [57]. It is important to note, however, that the reduced efficiency of the GSH-dependent system is accompanied by a more efficient Trx-dependent system. The maintenance of these two systems confirms the principle of mutual cooperation in the regulation of redox homeostasis in keratinocytes in the development of psoriasis. In addition, in the cytosol, the Trx-dependent system plays a dominant antioxidant role [59], suggesting that it provides reducing conditions for keratinocyte proteins both in the development of psoriasis and during UV therapy. However, since the development of psoriasis promotes both a decrease in the activity of glutathione peroxidase and catalase and the level of vitamin E directly involved in the protection of membrane phospholipids, the intensification of the lipid peroxidation process seems evident under these conditions.

Moderate oxidative stress may be beneficial in combating specific pathologies [60]. However, in order to eliminate the metabolic consequences of excessive oxidative stress during psoriasis and phototherapy—especially with respect to unchanged cells—compounds that modify redox conditions are actively sought after. In this study, the therapeutic effect of CBD, a natural compound with anti-inflammatory and antioxidant properties, was assessed [18,61].

For CBD to be topically therapeutic, it must effectively penetrate skin cells. Our results confirm that when entering keratinocytes, CBD—especially after exposure to UVA radiation—accumulates mainly in keratinocyte membranes, particularly in keratinocytes of patients with psoriasis. This is probably the result of the lipophilic nature of CBD and modification of the biomembrane structure under oxidative conditions [62]. The transportation of CBD through membranes can be facilitated by the action of membrane transporters (ABC transporters) shuttling endogenous and exogenous compounds [63]. This study indicates that although the development of psoriasis promotes the activation of the BCBP transporter, UV radiation, especially UVB, also activates MDR and MRP transporters. Literature data confirm that the oxidative stress we observed in psoriasis and after UV irradiation increases the activity of transmembrane transporters (MRP, MDR, and BCRP) in keratinocytes [64]. However, CBD treatment significantly reduces the activity of BCRP and MDR transporters. Studies on mouse brain cells have shown that MDR and BCRP knockout animals did not display perturbed CBD transport, but CBD inhibited both MDR and BCRP transporters [65]. In contrast, CBD can induce MRP expression in C57BL/6J mice hepatocytes through activating the redox-sensitive transcription factor Nrf2 [66], a notion that is consistent with our previous observations [30]. However, regardless of the transportation across cell membranes via membrane transporters, CBD can also be transported through voltage-dependent anion channels (VDAC) [67]. Based on the results of this study, it is difficult to unambiguously determine which membrane mechanisms facilitate the penetration of keratinocytes by CBD. Undoubtedly, CBD can penetrate the epidermal cells. Its metabolic activity in these cells is of great importance because it belongs to the group of cannabinoids involved in modeling the level of oxidative stress.

The findings of this work show that CBD, by reducing the activity of ROS-generating enzymes, reduces ROS levels, especially in keratinocytes from both healthy and patient cells treated with UV radiation. However, in the keratinocytes of patients with psoriasis, the ROS level, even after CBD treatment, is still elevated, especially when these cells have been exposed to UVB radiation. This observation is important because the skin is exposed to sunlight containing UV radiation on a daily basis, and also because phototherapy—which aims to remove disease-modified cells—is used in the treatment of psoriasis [68]. Previous reports confirm the ability of CBD to inhibit the production of ROS in oligodendrocyte progenitor cells through reducing the activity of the enzymes responsible for their generation [69]. Moreover, it is known that CBD, like other antioxidants, can also interrupt free radical chain reactions, capturing them, or transforming them into less active forms [70]. CBD also reduces ROS generation by chelating transition metal ions involved in the Fenton reaction to form extremely reactive hydroxyl radicals [71].

In addition to directly lowering the level of oxidants, CBD also modifies the redox balance by increasing the level/activity of antioxidants. The results of this study suggest that CBD increases the efficiency of the GSH-dependent system in the keratinocytes of healthy people, while it reduces the effectiveness of this system in the keratinocytes of patients with psoriasis and after UV irradiation. This can be explained by the high affinity of CBD for GSH cysteine and GSH-Px selenocysteine [30], especially in the case of the more elevated CBD levels in psoriasis cells. It has also been suggested that the reactive metabolite of CBD—cannabidiol hydroxyquinone—reacts covalently with cysteine to form adducts, for example, with glutathione and cytochrome P450 3A11, thus inhibiting their biological activity [72]. CBD, on the other hand, supports the action of antioxidant enzymes such as glutathione peroxidase, preventing the reduction of tin ions necessary for the biological activity of this enzyme, the level of which is usually reduced in pathological conditions [73]. CBD increases the level/activity of Trx and TrxR in the keratinocytes of healthy people. It has been previously shown that CBD—by increasing the transcriptional activity of Nrf2—leads to an increase in the level of thioredoxin and thioredoxin reductase, which indicates that the thioredoxin system is necessary for the survival of keratinocytes [74]. In addition, CBD has a stabilizing effect on catalase activity as well as the level of lipophilic vitamins, by preventing them from oxidizing. CBD possesses much higher antioxidant abilities (30%–50%) than α-tocopherol or vitamin C [75].

The results of this study highlight a distinct response of keratinocytes from patients with psoriasis from cells from healthy individuals, especially in the context of UV cell irradiation. The ability of CBD to partially prevent redox imbalance, particularly in the cells of healthy individuals, indicates a potential protective action of CBD on the components of cells susceptible to oxidative conditions, including phospholipids and proteins.

### 4.2. Phospholipid Metabolism in Keratinocytes Treated with CBD

Epidermal cells, including keratinocytes, are rich in phospholipids, which not only contribute to the formation and maintenance of the epidermal barrier, but also play a key role in maintaining the metabolic functions of cells [76]. The level of phospholipids, as well as products of their ROS-dependent and enzymes-dependent metabolism, such as lipid peroxidation products and endocannabinoids, is regulated by redox conditions, but these phospholipid derivatives are also themselves involved in regulating cellular redox balance [9,77].

Oxidative conditions, which are observed in keratinocytes of patients with psoriasis, promote oxidative modifications of phospholipids, as shown by increased levels of the lipid peroxidation products 8-isoprostanes and 4-HNE. 4-HNE is a highly reactive electrophilic aldehyde that readily reacts with nucleophilic groups. Therefore, 4-HNE can react with lipids, nucleic acids, peptides, and proteins, particularly those that contain cysteine, histidine, and lysine residues. As a result, 4-HNE can modify the structure of most antioxidant proteins and peptides involved in the protection of biomembranes, including components of GSH and Trx-dependent systems, as well as catalase [58]. Additionally, the level of 4-HNE-protein adducts is increased in the keratinocytes of psoriatic patients. The formation of 4-HNE-protein adducts in psoriatic keratinocytes has already been demonstrated in proteomics studies, especially in the context of proteins involved in the antioxidant response [78]. The intensification of the lipid peroxidation process was also observed in the blood cells and plasma of patients with psoriasis vulgaris [49,50], which confirms that this disease, regardless of local metabolic modifications in skin cells, is also characterized by changes in systemic metabolism. The UV irradiation of keratinocytes also enhances lipid peroxidation by increasing the level of 4-HNE-protein adducts, promoting further metabolic changes. The formation of 4-HNE-protein adducts enables 4-HNE to participate in the multi-step regulation of metabolic pathways (including signaling pathways associated with activation of kinases), as well as transcription factors that are responsible for redox homeostasis (such as Ref-1, Nrf2, p53, NFκB, and Hsf1). The formation of 4-HNE-protein adducts also affects the generation of pro-inflammatory factors and anti-apoptotic proteins [58,79]. Moreover, UV radiation impacts epidermal cells, which respond via a cascade of inflammation markers, such as cytokines (IL-6, Il-8, TNFα) and 8-isoprostanes that can be used as an indicator of photooxidative damages to cells and the extracellular matrix [80].

CBD—by reducing the level of ROS—reduces the likelihood of oxidative phospholipid modifications. This confirms the observed decrease in the level of 4-HNE and 4-HNE-protein adducts, especially in the case of keratinocytes from patients with psoriasis, as well as a decrease in the level of 8-isoprostane, especially in the keratinocytes of healthy people. Previous reports also confirm the effectiveness of CBD in preventing lipid peroxidation [81]. CBD has been observed to reduce MDA and 4-HNE levels in mice hippocampal cells (HT22) without oxygen and glucose under reperfusion conditions, as well as in liver homogenates of C57BL/6J mice [81,82]. CBD also reduced MDA and 4-HNE levels in mice with diabetic cardiomyopathy and in the livers of mice in the acute alcohol intoxication model [21,82]. CBD showed evidence of decreasing the level of isoprostanes in the cortex of transgenic mice modeling Alzheimer’s disease [83]. Since the effect of reactive lipid peroxidation products (particularly 4-HNE) on cellular metabolism is concentration-dependent [58], CBD—by reducing the severity of lipid peroxidation—may promote protective mechanisms in keratinocytes. In this situation, 4-HNE, by inducing protein synthesis or activity, may modify signal transduction (e.g., through stimulating kinases), and can, therefore, exert physiologically beneficial effects that promote cell survival and proliferation [58].

Regardless of lipid peroxidation, enzyme-dependent phospholipid metabolism leads to the formation of another group of phospholipid derivatives—endocannabinoids, which are involved in both the physiology and pathology of skin cells, including psoriasis and response to UV-irradiation [84]. This is consistent with the results of this study. The level of endocannabinoids depends mainly on the activity of the enzymes involved in their metabolism, such as FAAH and MAGL. The levels of FAAH and MAGL tend to increase in psoriasis and after the UV irradiation of keratinocytes, as demonstrated by our findings. Endocannabinoid levels may also be changed by the decrease in the activity of the ABC transporters (multidrug resistance protein 1—MDR1—and breast cancer resistance protein—BCRP), which is implied by the results of this study. Previous literature data also indicate that the anandamide transporter (AMT) is responsible for AEA uptake in cells, including keratinocytes [85]. In keratinocytes of patients with psoriasis and UV-irradiated, AEA and 2-AG levels are reduced and PEA levels are increased. However, CBD tends to reduce AEA levels in control keratinocytes and to increase AEA levels in UV-irradiated keratinocytes from patients. A previous report has shown that CBD modulates the function of the endocannabinoid system by increasing anandamide levels in human keratinocytes (HaCaT) [86]. Other data, however, indicate that CBD inhibits AEA cellular uptake and catabolism, competing with AEA for binding to fatty acid binding proteins (FABPs) responsible for the transport of hydrophobic compounds in the cytosol [87], but not for inhibiting human FAAH as suggested earlier [88]. Our results confirm the lack of CBD effect on FAAH/MAGL activity in the keratinocytes of healthy people and patients with psoriasis but show a decrease in their activity after UV irradiation. Furthermore, the treatment of CBD irradiated with UV keratinocytes significantly reduces PEA levels, especially in patients with psoriasis.

The metabolic effects of endocannabinoids are mainly associated with the activation of membrane receptors, including CB1/2, TRPVs, PPARs, for which CBD is also a ligand [14,23]. Thus, CBD may affect cellular metabolism by modulating endocannabinoid agonist activity or by directly competing with endocannabinoids in activating/antagonizing/inhibiting cannabinoid receptors in a concentration-dependent manner [89,90]. However, given the lack of effect of CBD on the level of major endocannabinoids in patients with psoriasis, and its modification of receptor expression, direct CBD interaction with the receptors is most likely. The activation of the CB1 receptor (for which CBD is a weak agonist [91]) in keratinocytes of patients with psoriasis, as well as after UV irradiation, may increase ROS production and the response of pro-inflammatory TNFα synthesis [8]. Moreover, CBD—by activating CB1 in other epidermal cells (melanocytes)—increases melanogenesis and tyrosinase activity [92]. A stronger increase in CB2 receptor expression was observed in both keratinocyte groups (from healthy subjects and patients) treated with CBD, while AEA and 2-AG levels were reduced. This confirms that CBD is a real CB2 receptor agonist. Its action could lead to a decrease in the level of ROS and TNFα, and thus contribute to the reduction of oxidative stress and inflammation [93]. Therefore, the overall response of cannabinoid receptors indicates that CBD has stronger antioxidant activity compared to pro-oxidative. However, when UV-irradiated cells are treated with CBD, the total effect of activation of CB1/2 receptors indicates that pro-oxidative conditions are promoted in patient keratinocytes. Another molecular target of CBD is the vanilloid receptor (TRPV1) [89], the primary ligand of which is PEA, which exhibits significantly increased levels in the keratinocytes of patients and in cells after UV irradiation. Thus, CBD can also indirectly modify the activation of CB1 and CB2 receptors through AEA and TRPV1 [91]. Published literature data indicate that PEA may reduce the expression and the levels of inflammatory cytokines in skin diseases through this mechanism [86]. Accordingly, PEA, at higher levels, may behave as an endogenous protective agent against UV-induced inflammation. This effect may contribute to the well-known therapeutic effect of UV radiation in chronic inflammatory skin diseases. Therefore, the assessment of the protective effect of CBD on keratinocytes of healthy people, and the destructive effect that could lead to apoptosis of keratinocytes of patients with psoriasis, requires further research, including the simultaneous assessment of inflammation.

Oxidative stress observed in the keratinocytes of patients with psoriasis is a critical element of the immune response to the pathology, further enhanced by UV radiation. Metabolic modifications, especially of membrane phospholipid/protein, can lead to excessive apoptosis or to the modulation of cells signaling dependent on phospholipid derivatives that are partly responsible for the modulation of oxidative stress. Therefore, CBD treatment, which leads to the accumulation of CBD mainly in keratinocyte membranes, particularly UV-irradiated psoriasis cells, reduces oxidative phospholipid modifications and their consequences. At the same time, CBD, by increasing the level of PEA, can contribute to reducing inflammation. The observed changes may indicate the cellular mechanism of CBD action in psoriasis and in the case of the UV radiation of a patient’s skin.
